# Flight to insight: maximizing the potential of *Drosophila* models of C9orf72-FTD

**DOI:** 10.3389/fnmol.2024.1434443

**Published:** 2024-06-10

**Authors:** Nicole A. d’Almeida, Marla Tipping

**Affiliations:** Providence College, Providence, RI, United States

**Keywords:** frontotemporal dementia (FTD), *Drosophila melanogaster*, neurodegeneration, C9orf72, metabolism, circadian rhythm

## Abstract

Advancements in understanding the pathogenesis of C9orf72-associated frontotemporal dementia (C9orf72-FTD) have highlighted the role of repeat-associated non-ATG (RAN) translation and dipeptide repeat proteins (DPRs), with *Drosophila melanogaster* models providing valuable insights. While studies have primarily focused on RAN translation and DPR toxicity, emerging areas of investigation in fly models have expanded to neuronal dysfunction, autophagy impairment, and synaptic dysfunction, providing potential directions for new therapeutic targets and mechanisms of neurodegeneration. Despite this progress, there are still significant gaps in *Drosophila* models of C9orf72-FTD, namely in the areas of metabolism and circadian rhythm. Metabolic dysregulation, particularly lipid metabolism, autophagy, and insulin signaling, has been implicated in disease progression with findings from animal models and human patients with C9orf72 repeat expansions. Moreover, circadian disruptions have been observed in C9of72-FTD, with alterations in rest-activity patterns and cellular circadian machinery, suggesting a potential role in disease pathophysiology. *Drosophila* models offer unique opportunities to explore these aspects of C9orf72-FTD and identify novel therapeutic targets aimed at mitigating neurodegeneration.

## Introduction

Frontotemporal dementia (FTD) comprises a spectrum of rare neurodegenerative disorders resulting from neuronal damage in frontal and temporal lobes of the brain (Surguchov, 2021). It is marked by progressive deterioration in executive functioning, behavior, language, and personality. Specific patterns of cognitive and behavioral deficits characterize the three FTD subtypes: behavioral variant FTD (bvFTD), which is most common, and nonfluent and semantic variant primary progressive aphasia. Symptom onset typically occurs between ages 45 and 60 (DeJesus-Hernandez et al., 2011; Moore et al., 2020), making FTD one of the leading causes of early-onset dementia.

Despite advancements in understanding FTD, challenges remain in early and accurate diagnosis, as well as the development of effective disease-modifying treatments. Exploring the underlying molecular and genetic mechanisms is critical for understanding FTD pathophysiology and advancing therapeutic strategies.

## *Drosophila* as a model of FTD

### *Drosophila* model and C9orf72-associated FTD

*Drosophila melanogaster* models offer unique advantages for studying disease pathology and therapeutic intervention due to their short lifespan, fast reproduction, well-characterized genetics, and genetic similarity to humans. Flies have been particularly instrumental in neurodegeneration research, with their conserved gene pathology elements such as chaperone modification, protein aggregation, tissue deterioration, and behavioral characteristics (Bonini, 2022). These conserved properties combined with *Drosophila*’s robust genetic toolkit allow for manipulation of genes linked to FTD heritability.

The main heritable mutations are autosomal dominant and associated with three genes: chromosome 9 open reading frame 72 (C9orf72), progranulin (GRN), and microtubule-associated protein tau (MAPT). Deciding which genetic variant model to employ depends on the topic of interest, as the prominent aspects of the FTD phenotype and the geographical variability differ across variants (Greaves and Rohrer, 2019).

Across Europe and North America, the predominant genetic factor underlying FTD is a GGGGCC hexanucleotide repeat expansion in intron 1 of C9orf72 (DeJesus-Hernandez et al., 2011; Renton et al., 2011). The fundamental pathology involves both loss- and gain-of-function. Hypotheses on its mechanism include haploinsufficiency, creation of repeat RNA foci, and dipeptide repeat proteins (DPRs) formed via repeat-associated non-ATG (RAN) translation (DeJesus-Hernandez et al., 2011; Balendra and Isaacs, 2018). Notably, RAN translation leads to five distinct DPRs: poly-GA, poly-GP, poly-GR, poly-PA, and poly-PR (Ash et al., 2013; Zu et al., 2013).

Recent data reveals these three major hypotheses are not mutually exclusive. One study found that reduced C9orf72 activity increases susceptibility to degenerative stimuli, specifically glutamate-induced excitotoxicity and compromised DPR clearance (Shi et al., 2018). In essence, haploinsufficiency exacerbates toxic gain-of-function effects. This has been corroborated by similar studies establishing that C9orf72 reduction suppresses autophagy, resulting in DPR accumulation and neuronal death (Boivin et al., 2020; Zhu et al., 2020). Together, the literature offers that loss- and gain-of-function mechanisms synergize in C9orf72-associated pathology.

Introducing the C9orf72 mutation into the fly genome replicates key elements of the human FTD phenotype, facilitating exploration of cellular and molecular pathways implicated in C9orf72-associated neurotoxicity. Since *Drosophila* lack a C9orf72 homolog, studying loss-of-function is impeded but avoids risk of redundancy. By introducing this gene into flies, great strides have been made in exploring C9orf72-FTD.

### Current focus

Major mechanisms underlying C9orf72-FTD and insights from *Drosophila* have been extensively reviewed elsewhere (Balendra and Isaacs, 2018). Recent progress with fly models continues to focus primarily on RAN translation and DPRs; due to their role in C9orf72 hexanucleotide repeat expansions, factors regulating RAN translation are under investigation. Inhibited translation via interaction between ribosomal proteins and DPRs, specifically poly-GR and poly-PR, is proposed as a mechanism for DPR-induced toxicity in C9orf72-associated neurodegeneration. Therapeutic targeting of this translational repression shows promise, with studies indicating that translation initiation factor eIF1A expression can rescue toxicity (Moens et al., 2019). Additionally, the identification of Protein RPS25A, a component of the small (40S) ribosomal subunit required for RAN translation, suggests inhibiting this subunit as a promising therapeutic approach (Yamada et al., 2019).

There has also been interest in the post-translational modification of DPR’s and its contribution to FTD progression. One study identified three types of poly-GR arginine methylation: monomethylation, symmetric dimethylation, and asymmetric dimethylation. Analysis reveals both types of dimethylation reduce phase separation, with symmetric dimethylation occurring most frequently and correlating with longer disease duration. Therefore, poly-GR inclusion toxicity is influenced by methylation and correlates with clinical manifestations (Gittings et al., 2020).

## Latest advancements in the field

Newer focuses on *Drosophila* C9orf72-FTD highlight neuronal dysfunction, autophagy, and synaptic dysfunction. In terms of neuronal dysfunction, C9orf72 hexanucleotide repeat RNAs localize to dynamic mRNA transport granules in neurites, disrupting machinery responsible for transporting and translating mRNA. This results in transport granule dysfunction and branching defects, with two components modulating repeat toxicity: FMRP and Orb2 (Burguete et al., 2015). These findings successfully identify the involvement of transport granules in the disease pathology and their components as a potential therapeutic target.

Research on autophagy in *Drosophila* C9orf72 found that in motor neurons, 30-repeat DPRs disrupt the morphology and dynamics of the endoplasmic reticulum (ER), impairing autophagosome formation. Despite ER disruption in both axons and synapses, autophagosomes remained intact in axons, yet their biogenesis was hindered in synaptic termini (Sung and Lloyd, 2024).

Motor neurons can also be studied for synaptic dysfunction. A novel cell-autonomous excitotoxicity mechanism selectively associated with arginine-rich DPRs in glutaminergic neurons has been discovered using *Drosophila* C9orf72. These DPRs—poly-GR and poly-PR—were moderately toxic at 36 repeats, increasing synaptic boutons, activity zones, extracellular glutamate, intracellular calcium, and presynaptic NMDA receptor activation (Xu and Xu, 2018). This suggests neurodegeneration via glutamate excitotoxicity and synaptic overgrowth, which was presynaptic NMDA receptor-dependent and therefore cell-autonomous. More toxic 100-repeat DPRs result in loss of active zones, suggesting severe neurodegeneration via synaptic degeneration (Xu and Xu, 2018). These findings support glutamate inhibition therapies and warrant further investigation into synaptic dysfunction in C9orf72-FTD pathology.

## Research needs

### Metabolism

#### Metabolic model of neurodegeneration

Historically, medical research primarily emphasized understanding diseases’ molecular and genetic basis, targeting genes, proteins, and signaling pathways to uncover pathology and potential therapies. Cellular metabolism, though crucial, was not as extensively studied for its role in pathophysiology nor recognized as a primary driver of disease. However, since the late 20th and early 21st century, there has been a striking shift toward investigating metabolism in disease, spurred by technological advancements. Metabolic changes are now recognized as significant in disease development and progression, notably in cancer research, where metabolic reprogramming is a recognized hallmark of cancer cells (Hanahan and Weinberg, 2011). This trend has since expanded to other areas, such as autoimmune and endocrine disorders. Despite this, the emphasis on metabolism in neurodegenerative disorders has been strikingly less pronounced, especially in *Drosophila* models.

The lack of research is concerning, given insights from other animal models linking neurodegeneration and metabolism. C9orf72 involvement in lysosomal-autophagy pathway dysregulation has been robustly established via interaction with various proteins, including CARM1 involved in lipid metabolism (Liu et al., 2018), WDR41, SMCR8, Rab proteins, and the ULK1 complex involved in autophagy initiation (Sellier et al., 2016; Sullivan et al., 2016; Webster et al., 2016; Yang et al., 2016), and a master regulator of metabolism, mTOR (Amick et al., 2016; Ugolino et al., 2016; Ji et al., 2017). Further studies have connected C9orf72 neurodegeneration with altered lipid catabolism (Marian et al., 2023).

These findings are reflected in human patients with C9orf72 repeat expansion, where glucose metabolism is altered during clinical and preclinical disease stages (Cistaro et al., 2014; de Vocht et al., 2020; Popuri et al., 2021; Xia et al., 2023). Along with the distinct metabolic profile of the C9orf72 mutation, there is also a toxic decrease in metabolic flexibility regarding substrate transport and glycogen, adenosine, and fructose metabolism (Allen et al., 2019). This translates to impaired resilience to altered nutrient availability, energy demand, or environmental conditions, where cells struggle to effectively alternate fuel sources and adjust metabolic pathways to support cellular function and homeostasis. Together, these findings strongly support metabolic dysregulation as a pathogenic factor in C9orf72-related neurodegeneration.

#### *Drosophila* metabolism and disease

Compared to clinical research and other animal models, *Drosophila* research on this is sparse, however promising. Considerable potential can be seen in a recent study using C9orf72 to investigate autophagy and proteasome pathways. Results showed spironolactone, an aldosterone antagonist, effectively lowers DPR levels through the autophagy pathway. A similar reduction in DPR levels was achieved via the proteasome pathway using geldanamycin, an HSP90 inhibitor. The study also implicated cAMP and inhibition of protein kinase A (PKA) activity in reducing DPR, hinting at PKA inhibition’s therapeutic potential (Licata et al., 2022). Other research explores insulin/IGF signaling, revealing consistent impairment in C9orf72 repeat expansion flies. They observed a decrease in GGGGCC repeat and poly-GR toxicity with heightened insulin signaling, suggesting a neuroprotective role for insulin signaling (Atilano et al., 2021). Together, these fly models identify significant metabolic components in neurodegenerative pathogenesis and potential therapeutic avenues for C9orf72-FTD.

Like any model, flies present limitations, such as potential energy consumption differences due to physiological temperature disparities (Warr et al., 2018). This distinction arises because flies are ectotherms, while humans are endotherms. Nevertheless, the similarities are extensive. For instance, *Drosophila* fat body, oenocytes, malpighian tubes, proventriculus, and midgut parallel in function to human adipose hepatocyte, kidney, stomach, and intestinal tissue, respectively (Bharucha, 2009; Rajan and Perrimon, 2013; Warr et al., 2018). Flies also exhibit similar metabolic pathways and associated phenotypes, including insulin-signaling and lipolysis in diabetes and obesity, as well as conserved hormones, enzymes, and homeostatic mechanisms (Bharucha, 2009; Chatterjee and Perrimon, 2021). Just as fly metabolism has contributed to studies of other neurodegenerative diseases, such as Parkinson’s (Solana-Manrique et al., 2022) and Huntington’s disease (Aditi et al., 2016), these similarities support clinical relevance for fly metabolic studies.

#### *Drosophila* tools for studying metabolism

**Figure 1:**
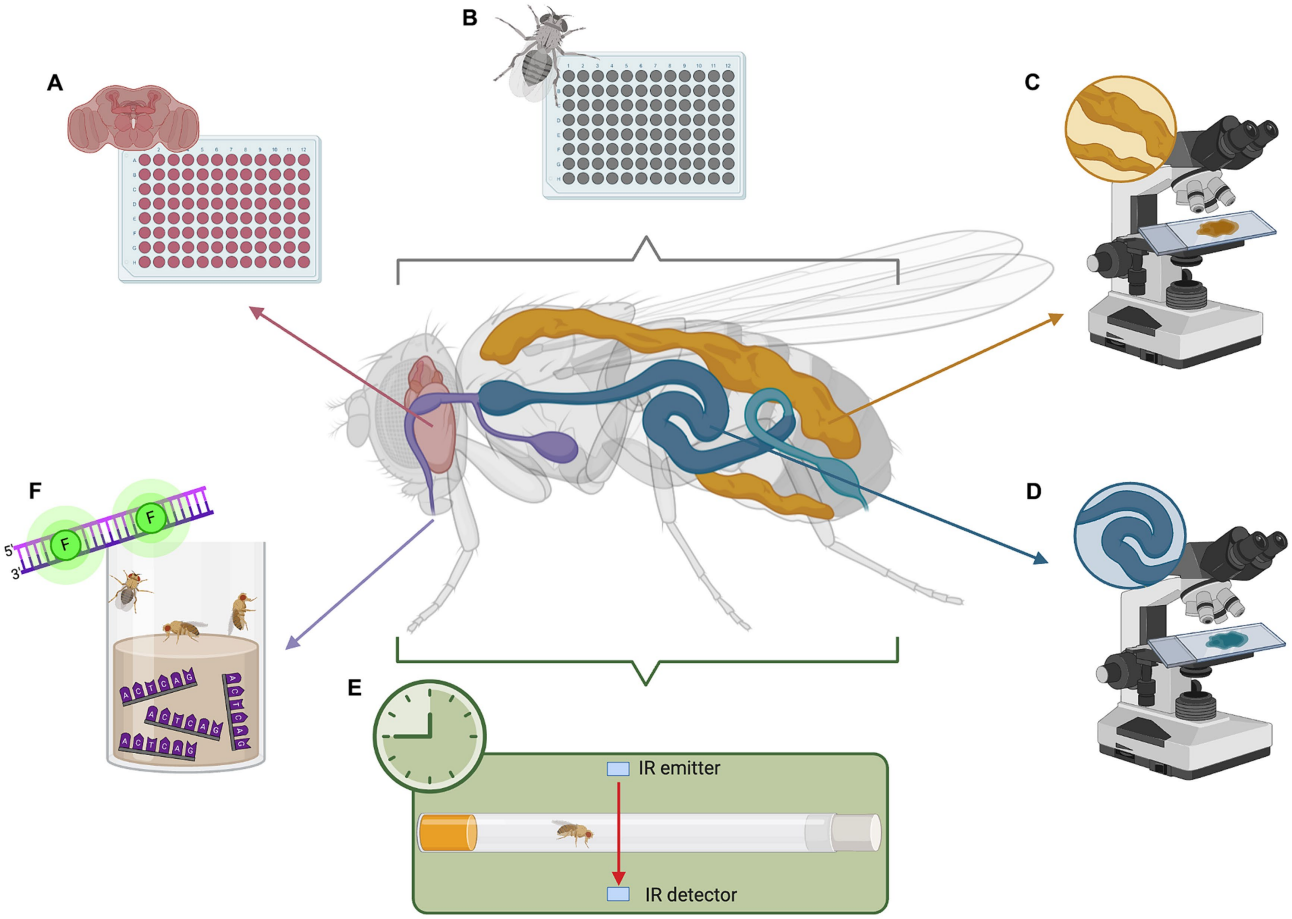
Analyzing metabolism and circadian rhythm in *Drosophila* model of C9orf72-FTD. **(A)** Whole-brain metabolic assays to measure oxygen consumption and extracellular acidification. **(B)** Whole-body metabolic assays to determine non-cell autonomous effects due to neurodegeneration. **(C)** Fat body staining to visualize lipid storage. **(D)** Gut staining to visualize microbiome and lipid digestion. **(E)** *Drosophila* activity monitor (DAM) to analyze activity and disruptions in circadian rhythm. **(F)** Barcode feeding assay to precisely measure food intake in a quantitative manner using qPCR. This image was created with BioRender (https://biorender.com/).

Various tools for studying fly metabolism are widely available, such as genetic manipulation, metabolic assays, locomotor activity assays, metabolomics, and imaging techniques. These methods are well-established and benefit from *Drosophila’s* unique advantages as a model organism, namely their short lifespan, fast reproduction, and well-characterized genetics. Widely used tools include tissue-specific RNAi, colorimetric triglyceride quantification, capillary feeder assays, lipid staining with Oil Red O, Sudan Black, and Nile Red, cholesterol extraction, glucose assays, luciferase-based ATP assays, and gas chromatography–mass spectrometry for metabolomic profiling (Figures 1B–D; Tennessen et al., 2014). More recently, barcode feeding assays have been developed (Park et al., 2018). This quantifies feeding history via qPCR of the fly body, measuring specific oligonucleotides that are incorporated into the food (Figure 1F).

Moreover, recent and ongoing research is lending to new techniques that facilitate further exploration. Of particular note is a novel method for simple, quick, and precise *ex vivo* whole-brain metabolic analysis (Figure 1A), enabling investigation of metabolism in small organisms in a tissue-dependent context (Neville et al., 2018). Adapted from the Agilent XFe96 metabolic analyzer, this method proves a useful tool for exploring fly metabolic dysfunction. Other methods have been optimized to dependably measure lactate, pyruvate, and 2-oxoglutarate *in vivo* using fluorescence resonance energy transfer (FRET) signals from metabolic sensors (Gándara et al., 2019). This toolkit presents an avenue for studying intact fly tissues and whole organs.

### Circadian rhythms

#### Circadian model of neurodegeneration

Circadian rhythms are observable in a plethora of organisms, as they are a defining feature across virtually all life forms. Initially overlooked, their significance in pathogenesis emerged with advancements in molecular biology and genetics. These 24-h cycles, governed by internal timekeeping mechanisms influenced by environmental, behavioral, and endogenous factors, impact metabolism, hormone, immune, and sleep–wake regulation. Disturbances in rhythmicity correlate with disease risk and severity (Fishbein et al., 2021) in a bidirectional manner. This has been validated for numerous pathologies, including immune (Liu et al., 2017; Shivshankar et al., 2020; Awuah et al., 2023), cardiovascular (Gottlieb et al., 2019; Hayter et al., 2021), psychiatric (Wehr et al., 1987; Meyer et al., 2024; Scheer and Chellappa, 2024), and neurodegenerative disorders (Fernández-Arcos et al., 2019; Sharma et al., 2020; Ibrahim et al., 2024). Given this wide reach, investigating the circadian dimension of disease is imperative.

This dimension of FTD has been robustly established in humans using sleep studies. Through actigraphy and sleep reports, FTD patients have been found to have reduced morning activity, total sleep, and sleep efficiency, and increased nighttime activity and sleep fragmentation (Anderson et al., 2009; Walker and Vaughn, 2021; Larsen et al., 2023). Unlike Alzheimer’s Disease, sleep disturbances were not reserved for those with severe cognitive and behavioral impairment, suggesting suprachiasmatic nucleus (SCN)-related neuropathology in FTD (Anderson et al., 2009).

In bvFTD, rest-activity rhythm alterations were time-locked to mornings and early afternoons (Filardi et al., 2024). Additionally, bilateral cortical thickness reductions in frontal brain regions were correlated with bvFTD patients’ prolonged sleep duration (Filardi et al., 2024). Given the role of cortical thickness in neurodegeneration and cognitive decline (Vidal-Pineiro et al., 2020; Subotic et al., 2021; Pletcher et al., 2023), these results imply a circadian influence in FTD pathogenesis.

C9orf72-FTD in particular is implicated in sleep disturbances, both at behavioral and cellular levels. Behaviorally, disruptive sleep events like restlessness and shouting have uniform associations with C9orf72-FTD but not GRN-FTD or MAPT-FTD (Sani et al., 2019). At the cellular level, circadian analysis of sleep/wake-associated cells in post-mortem human brain tissue with C9orf72-pathology reveals abundant DPR inclusions in pinealocytes and, to a lesser degree, SCN neurons (Dedeene et al., 2019). Via pineal gland dysfunction and the disturbed SCN-pineal gland axis, these findings suggest circadian implications in C9orf72-pathology.

Further, a potential connection between C9orf72 repeat expansions and Rapid Eye Movement Sleep Behavior Disorder (RBD) has been established. RBD is a sleep disorder considered to be an early indicator of neurodegeneration in Parkinson’s Disease, and, to a lesser extent, Amyotrophic Lateral Sclerosis (ALS) and FTD (Zhang et al., 2020). Among 344 RBD patients, researchers identified two carriers of C9orf72 repeat expansion with a risk haplotype associated with FTD (Daoud et al., 2014). As indicated by these findings, there may be a rare but potential link between these two disorders, suggesting C9orf72-FTD patients may be susceptible to sleep abnormalities.

#### *Drosophila* circadian rhythms and disease

Despite these outcomes, circadian studies are rare in C9orf72 animal models. Using *Drosophila*, researchers found sleep disruptions caused by poly-PR but not poly-GA DPRs. In poly-GR flies, altered sleep patterns included increased daytime sleep and decreased nighttime sleep, with nocturnal episodes elevated in number but reduced in duration, suggesting flies woke up more and slept less (Uy et al., 2024). These changes parallel the sleep disruptions in clinical patients (McCarter et al., 2016), suggesting a potential role of poly-GR in regulating sleep. This research highlights the pioneering potential of circadian research using C9orf72 *Drosophila* models.

As a highly conserved process, sleep regulation in flies resembles that of humans. Specifically, *Drosophila* exhibit all the fundamental characteristics of mammalian sleep (Hendricks et al., 2000; Shaw et al., 2000). This includes protein channel function, catecholamine wake-promoting effects, hypothalamic-like regulation, differential brain activity patterns, and the major clock genes *period* and *timeless* (Cirelli and Bushey, 2008; Dubowy and Sehgal, 2017). In mammals, the SCN regulates physiological and behavioral cycles and integrates external information, serving as the primary circadian pacemaker. Though *Drosophila* lack an SCN, they possess a functionally analogous set of approximately 150 neurons that regulate clock genes and behavioral activity (Dubowy and Sehgal, 2017). Overall, these similarities suggest clinical relevance of *Drosophila* circadian studies for human diseases.

#### *Drosophila* tools for studying rhythmicity

Years of research on *Drosophila* identified daytime activity and nighttime immobility, initially speculated as quiet wakefulness. In 2000, studies confirmed sustained nighttime immobility is a sleep-like state rather than quiet wakefulness (Hendricks et al., 2000; Shaw et al., 2000). This state exhibits an elevated, reversible arousal threshold and decreased responsiveness to external stimuli (Huber et al., 2004). It can last for hours, though a long duration is not a qualifying factor. Rather, sleep in *Drosophila* is operationally defined as at least 5 min of behavioral quiescence (Cirelli and Bushey, 2008).

Monitoring human circadian rhythms involves various methods, including actigraphy, body temperature, melatonin levels, hormone biomarkers, and rest-activity behavior (Yousefzadehfard et al., 2022). Sleep studies typically rely on recording eye movements and brain waves using electroencephalography (EEG) and polysomnography. These tools are adaptable to various animal models, such as pigs, dogs, rabbits, rats, and mice (Zong et al., 2023) but not to *Drosophila*. An alternative method uses local field potentials in brain regions involved in locomotion (Nitz et al., 2002; Qiu et al., 2021).

A common method used in flies is behavior-based paradigms. This includes actograms, temperature preference, courtship behavior, feeding behavior, or locomotor activity (Tataroglu and Emery, 2014). Locomotor activity is often measured using the *Drosophila* Activity Monitor (DAM) by Trikinetics. This houses a single fly in a locomotor tube where infrared light detects each time the fly breaks the beam, indicating its activity (Figure 1E). The DAM has been effectively utilized in studies (Chouhan et al., 2021; Karam et al., 2022), and various analysis software and protocols have been established (Chiu et al., 2010; Pfeiffenberger et al., 2010; Cichewicz and Hirsh, 2018; Silva et al., 2022). Thus, the DAM toolkit provides an effective means for studying circadian behavior in flies.

## Discussion

While progress has been made in unraveling FTD pathology, challenges remain in diagnosis and treatment, underscoring the importance of continued research. *Drosophila* are a powerful model for studying FTD pathogenesis, particularly in the C9orf72 mutation, a common genetic factor. By introducing this mutation into flies, key aspects of the human FTD phenotype are replicated, enabling exploration of cellular and molecular pathways implicated in C9orf72-neurotoxicity.

Historically, *Drosophila* studies focused on RAN translation and DPR toxicity in C9orf72-FTD pathology. Research then expanded to neuronal dysfunction, autophagy, and synaptic dysfunction, suggesting transport granule components and glutamate inhibition as possible therapeutic targets. Despite these advances, gaps in FTD pathogenesis, especially in metabolism and circadian rhythms, remain. Limited studies in *Drosophila* models show metabolic dysregulation, including altered lipid metabolism and autophagy, and circadian disturbances, urging further exploration. With evidence that metabolism is altered prior to symptomatic disease (Popuri et al., 2021), there is promise that metabolic biomarkers identified in *Drosophila* studies could be used for early diagnosis, and potentially preventative treatment.

Addressing these gaps is crucial for a comprehensive understanding of FTD. Considering that the fly community has accessible tools to study metabolism and circadian rhythms, investigating their ramifications on neurodegeneration is filled with potential. Exploring non-cell autonomous effects is also vital, as neuronal pathologies have systemic consequences. Leveraging the unique advantages of *Drosophila* models is crucial to advancing our understanding of FTD and uncovering novel therapeutic targets, ultimately benefiting FTD patients.

## Data availability statement

The original contributions presented in the study are included in the article/supplementary material, further inquiries can be directed to the corresponding author.

## Author contributions

NAD: Writing – original draft, Writing – review & editing. MT: Conceptualization, Writing – review & editing.
